# Real‐world epilepsy monitoring with ultra‐long‐term subcutaneous electroencephalography: A 15‐month prospective study

**DOI:** 10.1111/epi.18566

**Published:** 2025-07-19

**Authors:** Pedro F. Viana, Jonas Duun‐Henriksen, Andrea Biondi, Joel S. Winston, Dean R. Freestone, Andreas Schulze‐Bonhage, Benjamin H. Brinkmann, Mark P. Richardson

**Affiliations:** ^1^ Institute of Psychiatry, Psychology, and Neuroscience King's College London London UK; ^2^ Epilepsy Centre King's College Hospital NHS Foundation Trust London UK; ^3^ Centro de Estudos Egas Moniz, Faculdade de Medicina Universidade de Lisboa Lisbon Portugal; ^4^ UNEEG Medical Lillerød Denmark; ^5^ Graeme Clark Institute University of Melbourne Melbourne Victoria Australia; ^6^ Epilepsy Center, Department of Neurosurgery University Medical Center Freiburg Freiburg Germany; ^7^ Bioelectronics Neurology and Engineering Laboratory, Department of Neurology, Department of Physiology and Biomedical Engineering Mayo Clinic Rochester Minnesota USA

**Keywords:** epilepsy, mobile health, seizure detection, subcutaneous EEG

## Abstract

**Objective:**

Novel subcutaneous electroencephalography (sqEEG) systems enable prolonged, near‐continuous cerebral monitoring in real‐world conditions. Nevertheless, the feasibility, acceptability and overall clinical utility of these systems remain unclear. We report on the longest observational study using ultra‐long‐term sqEEG to date.

**Methods:**

We conducted a 15‐month prospective, observational study including 10 adult people with treatment‐resistant epilepsy. After device implantation, patients were asked to record sqEEG, to use an electronic seizure diary, and to complete acceptability and usability questionnaires. sqEEG seizures were annotated visually, aided by automated detection. Individualized temporal patterns of seizure occurrence were assessed via circadian circular statistics and via Fano factor analysis.

**Results:**

Over a median duration of 438 days, 10 patients recorded a median 18.8 h/day, totaling 71 984 h of real‐world sqEEG data. Adherence and acceptability remained high throughout the study. Although 754 sqEEG seizures were recorded across patients, more than half (52%) of these were not reported in the patient diary. Of the 140 (27%) diary reports not associated with an identifiable sqEEG seizure, the majority (68%) were reported as seizures with preserved awareness. The sqEEG to diary F1 agreement score was highly variable, ranging from .06 to .97. Patient‐specific patterns of circadian seizure occurrence and seizure clustering were found, including several relevant discrepancies between sqEEG and diary.

**Significance:**

We demonstrate feasibility and high acceptability of ultra‐long‐term (months–years) sqEEG monitoring. These systems help provide real‐world, more objective seizure counting compared to patient diaries. It is possible to objectively monitor individual temporal fluctuations of seizure occurrence.

**Key points**

- Ultra‐long‐term sqEEG monitoring was feasible and well‐accepted over a median of 438 days.
- Patients recorded a median of 18.8 h/day, totaling nearly 72 000 h of real‐world sqEEG data.
- Fifty‐two percent of sqEEG‐confirmed seizures were not reported in seizure diaries, showing diary underreporting.
- Patient‐specific seizure clustering and circadian periodicity patterns were found.

## 1 INTRODUCTION

In treatment‐resistant epilepsy, accurate identification of seizures and other epilepsy‐related objective disease information is a major challenge in clinical practice.^1^ Accumulated evidence demonstrates the unreliability of patient‐reported seizure diaries.^1^ Patients may underreport a large proportion of seizures, while overreporting some nonseizure symptoms as seizures. This can lead to inappropriate clinical management, from insufficient treatment exposing patients to seizure‐related harms to excessive treatment contributing to unnecessary side effects. Despite these limitations, seizure diary information remains the usual primary outcome measure for assessing efficacy of new treatments in clinical trials.^2^

Seizure unpredictability is also a major concern frequently highlighted in patient surveys.^3^ Although often perceived as completely random events, reports of nonrandom seizure timing date back millennia. Sir William Gowers classified epilepsy “chronotypes” (“nocturnal,” “diurnal,” and “mixed”)^4^ and also described seizure clusters, or “groups of attacks,” suggesting that “seizures beget seizures.”^4^ Seizure cycles are also being increasingly characterized with novel long‐term sensing, monitoring devices, and mobile apps.^5^, ^6^, ^7^

Tracking objective disease information and reducing seizures have motivated the development of mobile health monitoring systems.^8^ Their clinical applications are wide‐ranging, from real‐time seizure detection alarms^9^ to offline accurate seizure counting^10^ and seizure forecasting.^11^, ^12^, ^13^ Key requirements for the adoption of these systems include evidence that the technology is reliable, has suitable performance characteristics, addresses patient needs, and is usable in the long term.^14^

Available seizure detection devices mostly monitor indirect, noncerebral biosignals as proxies for mostly major motor (tonic–clonic) seizures.^9^, ^14^ Electroencephalography (EEG) remains the most important instrument in the evaluation of epilepsy, with the ability to characterize multiple seizure types, detect interictal epileptiform activity, and monitor sleep.^15^ However, scalp EEG is limited to a few weeks at most, due to the potential for skin injury, inconvenience of visible electrode wires, and signal quality degradation with time. Dry electrodes have been developed to overcome some of these limitations, but signal quality also tends to degrade.^16^ Behind‐the‐ear or in‐the‐ear EEG has been studied in small numbers of patients, but their long‐term signal quality is unknown.^17^, ^18^ Chronic invasive intracranial EEG systems have been developed as seizure warning systems^19^ or closed‐loop stimulation devices^20^, ^21^; however, they are associated with the risk of severe complications.

Subcutaneous EEG (sqEEG) could offer a trade‐off between minimal invasiveness/low risk and good signal quality.^22^, ^23^ sqEEG signal quality is similar to simultaneous scalp EEG^24^ and is highly stable over multiple months (ultra‐long‐term).^23^ Recent single cases and case series have reported on the system's feasibility, safety, and spectrum of clinical indications.^10^, ^25^, ^26^, ^27^, ^28^

We report here on the longest prospective study (to the best of our knowledge) using ultra‐long‐term sqEEG to date. We systematically assessed the system's usability and acceptability, the diagnostic yield for seizures of different types, its comparison to patient diaries, and utility to investigate individualized temporal dynamics of seizure occurrence.

## 2 MATERIALS AND METHODS

### 2.1 Study design and population

The SUBER study (Subcutaneous EEG: Forecasting of Epileptic Seizures Through Investigation of Long‐Term Dynamics of Seizure Occurrences, Stress, Sleep and Other Factors) was an observational, prospective, nonrandomized, and noninterventional study, conducted at King's College London and King's College Hospital NHS Foundation Trust. Participants' consent was obtained according to the Declaration of Helsinki, and the study was approved by the local ethics committee (19/LO/0354).

### 2.2 Patient selection

We aimed to recruit 10 adult people with treatment‐resistant epilepsy (>20 seizures per year according to seizure diary) of any syndrome, in which seizures were detectable by scalp EEG with two electrodes. We excluded patients with a diagnosis of potential seizure mimics (e.g., psychogenic nonepileptic seizures), significant medical comorbidities, or contraindication for placement of the subcutaneous EEG implant (e.g., planned magnetic resonance imaging [MRI] during the study period). The full inclusion/exclusion criteria can be found in Supporting Information S1. Patients were recruited from epilepsy clinics at King's College Hospital and St. George's University Hospital. New participants were recruited if a participant dropped out prematurely from the study.

### 2.3 Study procedures and data collection

A schematic of the main study procedures can be found in Supporting Information S2. Patients prescreened for eligibility criteria were approached during clinic or by a direct telephone call. Those interested in participating underwent an inclusion visit whereby eligibility criteria were confirmed, and preprocedure laboratory blood tests were performed.

Eligible patients were invited to a second visit during which the subcutaneous EEG device was implanted. Placement of the 24/7 SubQ implant involved a small 25‐mm incision made in the postauricular region using local anesthesia and sterile technique. The electrode was placed in the subgaleal space, oriented toward the expected site of the strongest ictal EEG activity (determined after examining the participant's previous investigations). More information about the implantation procedure can be found in Djurhuus et al.^29^

Data collection commenced 1–2 weeks after implantation. Patients were instructed to wirelessly connect the 24/7 EEG SubQ external logging device/data logger (Supporting Information S3), which supplies the implant with power and stores recorded sqEEG. Patients were asked to connect the data logger for as long as possible during the day and night, except in circumstances when the device could get wet (e.g., personal hygiene). A 15‐min data quality protocol was conducted to record background activity, as well as common artifacts (e.g., eye blinks, chewing, eye movements, head movements, walking, running), to later facilitate the visual interpretation of the EEG (example recording in Supporting Information S4).

Patients were asked to report their seizures using an electronic diary (Seer app, Seer Medical); they were given a Seizure Key card with description of each different seizure type. Diary entries were classified into three categories: nonconvulsive with preserved awareness, nonconvulsive with impaired awareness, and convulsive/tonic–clonic. Participants were also requested to use a commercial‐grade fitness tracker throughout the study (Fitbit Charge 3 or 4) that estimates heart rate, step counts, sleep duration, and sleep staging.

**FIGURE 1:**
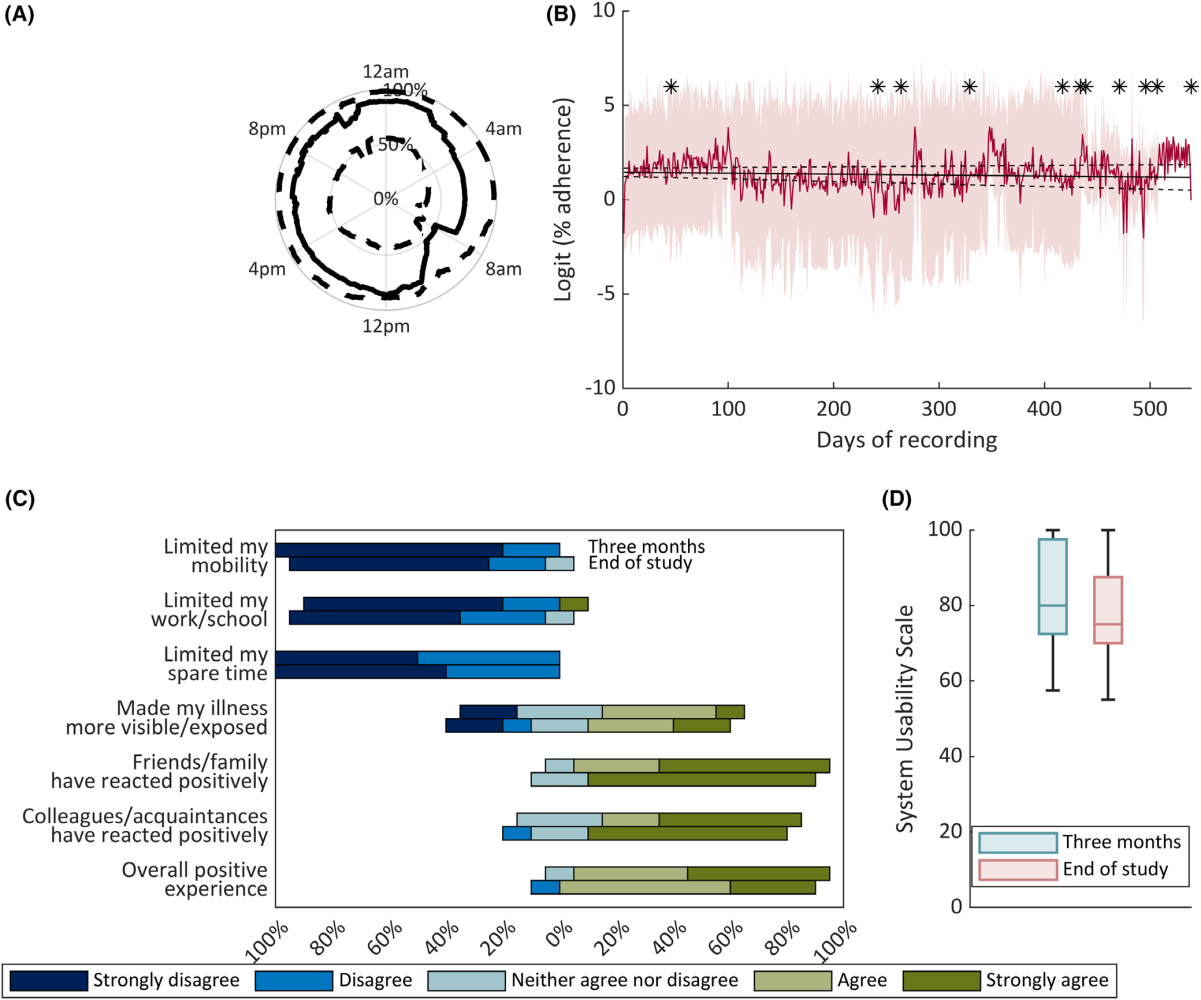
Adherence and acceptability of ultra‐long‐term subcutaneous electroencephalography in our cohort. (A) Circadian polar plot showing median (solid line) and interquartile ranges (dashed lines) of average adherence at different times of day, across study participants. (B) Long‐term adherence. Red line and shaded area represent the mean and SD of logit daily adherence, across participants. Black asterisks denote the end of the recording for each subject. The black solid line represents the result of the group‐level linear model, with intercept at 1.45 (equivalent to 81%), and dashed lines are 95% upper and lower bounds of the model. (C) Results of the acceptability questionnaires at 3 months and at study end. (D) System Usability Scale at 3 months and study end.

Up‐to‐monthly follow‐up visits were conducted to offload sqEEG data, review patient safety, experiences with the device, seizure diaries, and treatment changes. Patients completed the brief Illness Perception Questionnaire^30^ at baseline (Supporting Information S5). Participant satisfaction with the sqEEG system was measured at 3 months postimplantation and at study end. Overall satisfaction was assessed via a 7‐item Likert scale questionnaire (Figure 1C). Usability was assessed with a 10‐item System Usability Scale, which ranges from 0 to 100 (Figure 1D).^31^ Clinical care was not altered by participation in the study, hence medication and other treatment changes were allowed. Patients were encouraged to collect data, but no specific study adherence criteria for patient drop‐out were set. Device explantation at the end of the study was performed under local anesthesia during a half‐day hospital visit. Adverse events (AEs) and device deficiencies were collected throughout the study. The study procedures underwent several modifications throughout the study period (Supporting Information S6).

### 2.4 Data preprocessing and seizure annotations

The two‐channel subcutaneous EEG signal was recorded at a sampling rate of 207 Hz and bandpass filtered at .5–48 Hz with a finite‐impulse‐response equiripple design and 40‐dB attenuation filter, prior to review. The sqEEG was reviewed with dedicated software (UNEEG Episight viewer; example in Supporting Information S7) with an in‐built 10‐min spectrogram viewer and a high‐sensitivity data review reduction seizure detector. Two seizure detection algorithms were used in the study: (1) an initial version (v1.11) used in the first participant; and (2) an improved and published version (v2.0) used in the remaining participant data, which in a previous cohort has demonstrated a sensitivity of 86% and a false detection rate of 2.4 per day.^32^ Electrographic seizures were identified by a board‐certified electroencephalographer (P.F.V.) and an experienced EEG technologist (Christian Skaarup, UNEEG Medical). Several examples per patient were discussed and reviewed with a second board‐certified clinical neurophysiologist (J.S.W.). Patient‐specific electrographic seizure patterns (seizure signatures), taken from previous recordings, were initially assessed by reviewers to facilitate their identification on sqEEG. The sqEEG quality protocol recordings were also useful for identifying potential seizure mimics due to common rhythmic artifacts (e.g., movement artifact during walking). Electrographic seizures were further classified into convulsive or nonconvulsive, depending on whether clear convulsive (tonic followed by clonic) artifacts were seen, as previous preliminary work showed perfect interrater agreement to differentiate between these seizure types (Supporting Information S8).

The sqEEG data review process was conducted as follows: (1) review of events marked by the high‐sensitivity seizure detector, (2) review of periods around the patient diary reports (within 2 h pre‐ and postreport; this interval was chosen a priori as a reasonable balance between diary accuracy and data review burden), and (3) review of a random sample of 6‐h epochs (in 10‐min spectrogram epochs) comprising 10% of the whole recording. Finally, the full dataset (in 10‐min spectrogram epochs) was reviewed if sensitivity of the seizure detection algorithm was found to be <80%.

### 2.5 Statistical analyses

Descriptive analysis was summarized using median and interquartile range (IQR) for continuous variables and absolute count (percentage) for categorical variables.

Adherence to sqEEG was assessed at circadian, weekly, and long‐term timescales. A day‐of‐the‐week effect was investigated at the individual level via Kruskal–Wallis tests. To assess long‐term adherence to sqEEG, linear regression models were constructed, at both the group and individual levels. The outcome variable was percentage adherence per calendar day (after logit transformation), and the dependent variable was time (in days since start of recording).

Comparison to diary records was made by matching every sqEEG seizure with the closest diary event, if the event was reported in the vicinity (i.e., ±2 h) of the seizure. The F1 score was calculated to assess the overall agreement between diary events and sqEEG seizures.

Seizure clustering was assessed via inspection of cumulative seizure count plots, as well as by calculating the Fano factor (ratio of the variance to the mean of seizure frequency) across daily, weekly ,and monthly timescales, for both the diary and sqEEG.^33^, ^34^ For a Poisson process, the Fano factor = 1; a clustering process exhibits Fano factor > 1, and for a regular periodic process it is <1. Significance was calculated using a previously described method based on the gamma distribution.^33^, ^35^ Clustered seizures were defined as those preceded by at least one seizure in the past 24 h (see varying seizure cluster definitions in Haut^36^).

We analyzed the circadian (24 h) periodicity of both sqEEG seizures and diary events. We included patients with >10 seizures and/or diary events. We calculated the synchronization index (mean resultant vector length) to quantify the concentration of sqEEG or diary seizure occurrences across the 24‐h circadian cycle, with higher values indicating greater temporal clustering. To assess whether the distribution of seizure times deviated significantly from uniformity, we applied the omnibus test for circular data.^37^ Significance was assessed as *p*‐value < .05. Data analysis was performed using Microsoft Excel 365 and MATLAB (MathWorks, R2024a).

## 3 RESULTS

### 3.1 Study population

Among 15 patients who initially consented to participate in the study, two (SK, SO) failed screening and one (SL) withdrew consent before the implantation visit (details of the study recruitment workflow can be found in Supporting Information S9). Of the 12 patients implanted, two dropped out before recording usable data—one whose implant was misplaced during surgery, and with skin protrusion necessitating removal, and who did not wish to be reimplanted (SM), and one who reported an immediate headache and scalp pain postimplantation who did not wish to wait for improvement (SN). As previously mentioned, a device malfunction early in the study led to an early drop‐out of one participant (SB, recording 45 days), whereas another patient agreed to be reimplanted (SA → SA2). Hence, the final cohort with usable data included 11 datasets from 10 patients (Table 1). All patients had focal epilepsy of structural or unknown (i.e., MRI‐negative) etiology. Age ranged from 29 to 64 years, and half were male. All were on at least two regular antiseizure medications, and two participants also had active vagus nerve stimulation (SF, SI). The majority (7/11) of implant locations were left‐sided.

**TABLE 1:** Cohort clinical characteristics.

ID	Seizure types	Antiseizure medication	Etiology	Seizure focus on historical EEG
SA	FAS, FIAS, FBTCS	LEV, CBZ	Structural (LEAT)	Left temporal (+right temporal subclinical seizures on intracranial EEG)
SA2^a^	FAS, FIAS, FBTCS	LEV, CBZ, CLB^b^ ^,^ ^c^	Structural (LEAT)	Left temporal
SB	FAS, FIAS, FBTCS	CBZ, BRV, CLB^d^	Unknown	Right frontotemporal
SC	FIAS, FBTCS	CBZ, LMT, CLB, TPM,^b^ CLB^c^	Unknown	Right frontal
SD	FAS, FIAS	LEV, GBP, LMT	Unknown	Right frontotemporal
SE	FIAS, FBTCS	LMT, PRP, TPM, CLB^c^	Structural (congenital lesion + hippocampal sclerosis)	Left temporal
SF	FAS, FIAS, FBTCS	CBZ,^d^ LCM, VPA, CLB, LEV,^b^ (VNS)	Structural/immune (postencephalitis)	Left temporal (+right temporal subclinical seizures)
SG	FAS, FIAS, FBTCS	LMT,^d^ LEV, CLB^c^	Structural (subependymal heterotopia)	Left temporal
SH	FAS, FIAS	CBZ, LEV,^d^ BRV, PRP	Structural (childhood encephalomalacia + hippocampal sclerosis)	Left frontotemporal
SI	FAS, FBTCS	PHT, VPA, OXC, CLB,^c^ (VNS)	Unknown	Left centroparietal
SJ	FAS, FIAS, FBTCS	LCM, CBZ,^d^ LEV, TPM	Structural (FCD)	Right temporal

Abbreviations: BRV, brivaracetam; CBZ, carbamazepine; CLB, clobazam; EEG, electroencephalography; FAS, focal aware seizures; FBTCS, focal to bilateral tonic–clonic seizures; FCD, focal cortical dysplasia; FIAS, focal impaired awareness seizures; GBP, gabapentin; LCM, lacosamide; LEAT, low‐grade epilepsy‐associated neuroepithelial tumor; LEV, levetiracetam; LMT, lamotrigine; OXC, oxcarbazepine; PHT, phenytoin; PRP, perampanel; TPM, topiramate; VNS, vagus nerve stimulation; VPA, valproate.

^a^ Reimplantation of SA.

^b^ Started during study.

^c^ Pro re nata.

^d^ Stopped during study.

### 3.2 Device safety and device deficiencies

Twelve AEs occurred throughout the study, of which seven were deemed possibly or probably related to study participation (Supporting Information S10). Most AEs were mild and comprised of temporary pain/headache after the implantation procedure. Two serious AEs occurred throughout the study: one hospitalization due to community acquired pneumonia (unlikely to be related to study participation) and one unanticipated hospitalization to urgently remove a misplaced implant causing tip protrusion through the scalp (hospitalization was only necessary due to COVID‐19 restriction measures). All AEs were associated with recovery without any sequelae.

Thirty‐two device deficiencies (Supporting Information S11) occurred throughout the duration of the study, of which 25 affected sqEEG data collection. Most deficiencies were related to the external data logger, only temporarily affected data collection, and were resolved by rebooting or (more rarely) replacement of the devices.

### 3.3 Device adherence and acceptability

For a median recording duration of 433 days, participants recorded a median of 18.8 (IQR = 12.1) h per day (i.e., 78.4% of the recording time; Table 2). Five participants recorded >20 h/day, whereas three recorded <12 h/day. One participant (SI) had simultaneous implantation of a vagus nerve stimulation device and experienced unanticipated freedom from impaired awareness seizures, which limited his motivation to record. Overall, 71 984 h of real‐world sqEEG were collected.

**TABLE 2:** Implantation and recording characteristics of the cohort.

Subject ID	Implant location (approximate overlap with 10–20 system)	sqEEG recorded, h	Recording duration, days	Data capture rate, h/day, %	Seizures recorded, *n*
SA	Left temporal (F7‐T3)	4941	241	20.5, 85.4%	33
SA2^a^	Left frontotemporal (F3‐T3)	9319	495	18.8, 78.4%	92
SB	Right temporal (F8‐T4)	809	45	17.9, 74.7%	36
SC	Right frontotemporal (F4‐T4)	6943	506	13.7, 57.2%	56
SD	Right frontotemporal (F4‐T4)	10 682	470	22.7, 94.7%	54
SE	Left frontotemporal (F3‐T3)	11 692	538	21.7, 90.5%	54
SF	Left frontotemporal (F3‐T3)	10 363	438	23.7, 98.6%	132
SG	Left frontotemporal (F3‐T3)	2783	263	10.6, 44.1%	203
SH	Left frontotemporal (F3‐T3)	3620	433	8.4, 34.8%	31
SI	Left centrotemporal (C3‐T3)	1360	329	4.1, 17.1%	0
SJ	Right frontotemporal (F4‐T4)	9472	417	22.8, 95.0%	63
Total	–	71 984	–	–	754
Median (IQR)	–	2783 (6943–10 363)	433 (263–495)	18.8 (12.1), 78.4% (44.1%–94.7%)	54 (33–92)

Abbreviations: IQR, interquartile range; sqEEG, subcutaneous electroencephalography.

^a^ Reimplantation of SA.

Circadian adherence plots (Supporting Information S12) showed highly individualized patterns of adherence, from strictly fixed hours off recording in some participants, to a preference to record during either the day or night in others. A weekday effect was statistically significant in four participants (Supporting Information S13). Analyzing long‐term adherence, a group‐level model showed no significant attrition throughout the study (Figure 1B), and most individual long‐term adherence models also showed a temporal trend close to zero (Supporting Information S14).

Acceptability and usability of the system was overall high and remained high throughout the study (Figure 1C,D). In general, participants felt that the system did not limit their daily lives. Approximately half of participants felt that the system made their chronic illness more exposed; however, the general impression from family, friends, or colleagues was positive. Specific comments about device acceptability and usability can be found in Supporting Information S15.

### 3.4 Recorded seizures and comparison to diary

**FIGURE 2:**
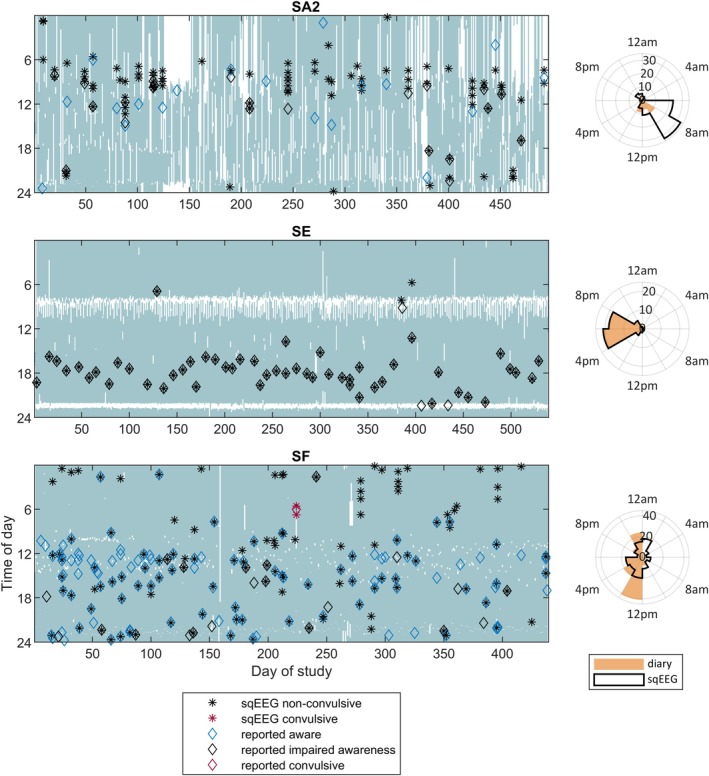
Example participant recordings. Left panel: Day‐of‐study versus time‐of‐day plots. The shaded areas indicate periods when the patients were recording. Diamonds represent times of diary‐reported aware (blue), impaired awareness (black), and convulsive (red) seizures. Asterisks indicate times of nonconvulsive (black) and convulsive (red) subcutaneous electroencephalographic (sqEEG) seizures. SA2 shows examples of clustered seizures (vertical columns of multiple asterisks), as well as a mixture of well‐reported with missed sqEEG seizures. Note the circadian (morning) preference for seizure occurrence. SE is an example of a very strict device adherence pattern, perfect diary‐to‐sqEEG agreement, absence of seizure clustering, and circadian (early evening) preference for seizure occurrence. SF shows many reported (mostly aware) events without an sqEEG correlate, many nonreported nocturnal sqEEG seizures, and one nonreported clustered tonic–clonic sqEEG seizure. Right panel: Circadian polar plots of sqEEG seizures (black outlined) and diary‐reported (orange shaded) occurrence for each corresponding participant.

Example recordings comparing sqEEG and diary events can be found in Figure 2. After final data review, 754 seizures were annotated in total across participants (Table 2). The number of seizures per participant ranged from zero (SI, with low adherence and reported freedom from impaired awareness seizures) to 203. A small number of convulsive seizures (*n* = 10) was recorded in three participants. Each patient (and seizure type) had their own ictal seizure signature visible on the EEG time series and spectrogram (examples in Supporting Information S16).

Conversely, 592 events were reported in participants' seizure diaries, of which 506 were reported at times when the sqEEG device was being used. Most diary reports were reported as nonconvulsive impaired awareness (*n* = 237) and nonconvulsive aware seizures (*n* = 236).

After matching individual sqEEG seizures with the closest diary events (within 2 h from each other), we found that more than half (52%) of recorded sqEEG seizures were not reported in the diary, including three convulsive seizures (Table 3). Conversely, 140 diary events were not associated with a sqEEG seizure. Most (68%) nonrecorded diary events were reported as aware seizures, and no reported convulsive seizures were missed by the sqEEG device. In addition, there were discrepancies between the type of seizure reported in the diary and the pattern detected on sqEEG. Across patients, the F1 agreement score between reporting a diary event and recording a sqEEG seizure was .58, and the within‐patient median F1 score was .56 (IQR = .40–.68), ranging from .06 (SI) to .98 (SE).

**TABLE 3:** Comparison between diary‐reported events and sqEEG‐recorded seizures.

Diary	sqEEG			Total
	Nonconvulsive	Convulsive	Not recorded	
Aware	141	0	95	236
Impaired awareness	201	1	35	237
Convulsive	3	6	0	9
Unclassified	14	0	10	24
Unreported	385	3	–	388
Total	744	10	140	

Abbreviation: sqEEG, subcutaneous electroencephalography.

### 3.5 Patterns of seizure occurrence

**FIGURE 3:**
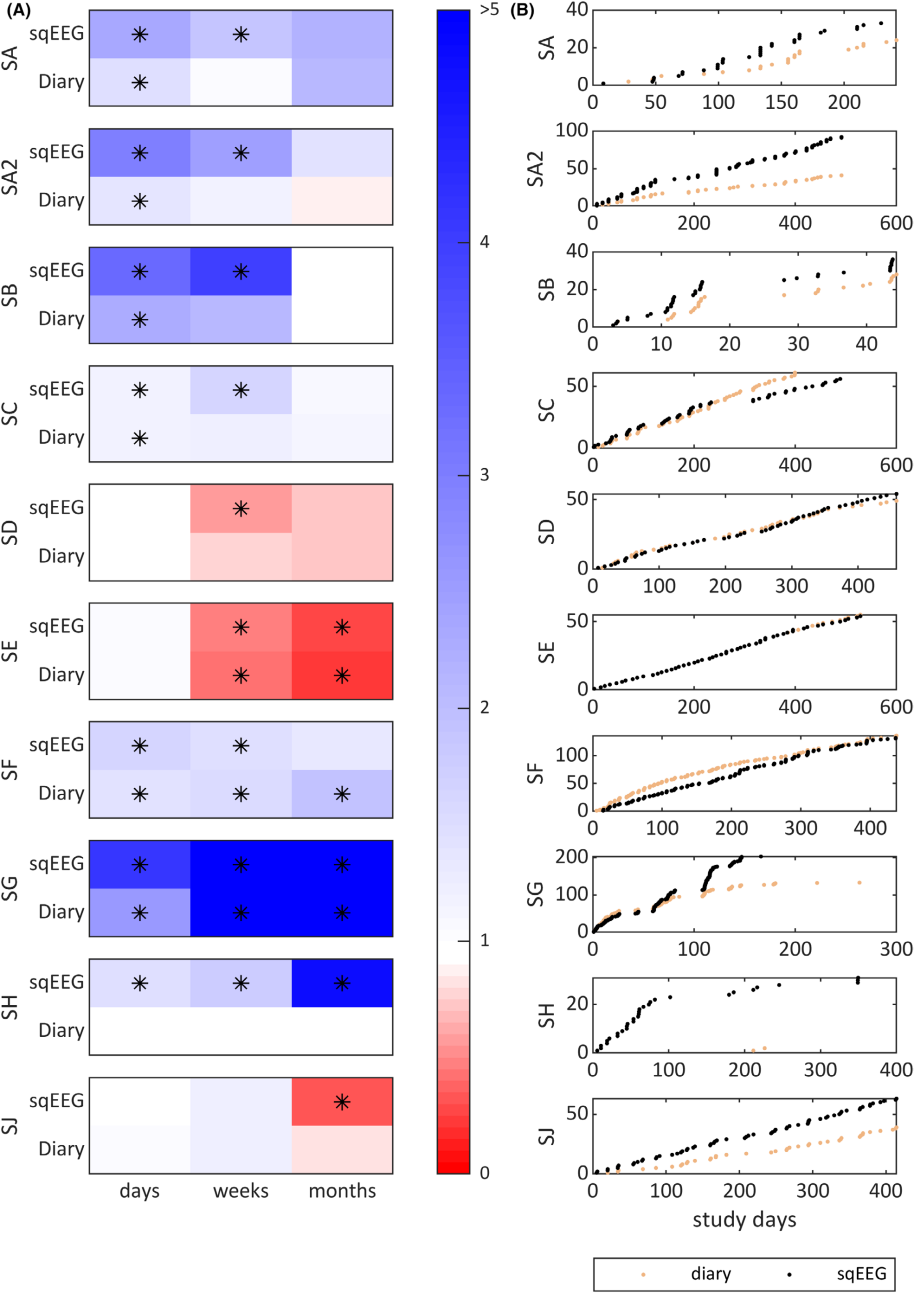
Seizure clustering in the SUBER cohort. (A) Fano factor (FF) values for each participant at day, week, and month timescales, based on the subcutaneous electroencephalographic (sqEEG) and diary data. An FF of 1 corresponds to an expected Poisson distribution. Asterisks indicate significant (*p* < .05) deviation from FF = 1. Note, for example, FF values > 1 in SA, SB, and SA2, suggesting a high degree of clustering, and FF < 1 in, for example, SE, suggesting a more periodic nonclustering pattern. (B) Scatter plots for the different participants showing seizure occurrence versus cumulative seizure counts, for diary (orange) and sqEEG (black) data, showing clustering patterns in some (SA, SA2, SB, SG) participants compared to others without clustering (SD, SE, SF).

The proportion of clustered seizures (seizures occurring within 24 h of a preceding seizure) ranged from 2% (SD) to 87% (SG), with a median of 42%. In addition, a subset of participants had increased Fano factor values consistent with seizure clustering at different timescales (Figure 3A). Conversely, some participants (e.g., SE) had Fano factor values < 1, in keeping with a periodic pattern of seizure occurrence. Fano factors were more frequently significant when analyzing the sqEEG, compared to the diary (Figure 3A).

Analyzing circadian seizure periodicities (Supporting Information S17), most participants included in the analysis had significant circadian cycling of seizure occurrence, evident from either the seizure diary or sqEEG. In several participants, there was a marked similarity between diary and sqEEG seizure distributions over the time of day. However, some discrepancies were found. For example, in SF the diary suggested a strong 24 h cycle, whereas the sqEEG data showed no significant periodicity. Analyzing the participant's recording (Figure 2C), it was evident that the patient was not reporting many nocturnal seizures, which likely led to an overestimation of a diurnal preference for seizure occurrence.

### 3.6 Performance of automated seizure detector

Supporting Information S18 shows the performance of the employed seizure detection algorithm aimed at reducing the amount of data needing manual review. Performance was quite variable between participants, with a median (IQR) sensitivity of 70.5% (48%–94.6%) and prereview false detection rate of 3.4/day (3–15.8/day). Performance was excellent in a group of patients (e.g., SC, SE, SJ) but poor in others (e.g., SB, SD, SF), which required full review of their datasets.

## 4 DISCUSSION

We conducted a systematic prospective observational study using ultra‐long‐term sqEEG, recording >70 000 h of EEG from 10 patients, spanning up to 15 months each, and including >700 confirmed seizures. We demonstrate the feasibility of ultra‐long‐term sqEEG monitoring, and we highlight important aspects of clinical applicability of these systems, namely the improvement in documentation of different seizure types, aided by automated seizure detection, and insights into personalized long‐term temporal dynamics of seizure occurrence.

The implantation of the sqEEG device was well tolerated by most patients in the study. Most side effects were mild, transient, and related to the local anesthetic procedure. However, there was one significant complication after surgery (electrode erosion through the skin), and one patient dropped out early due to localized headache. Early malfunction of the sqEEG device led to an additional study drop‐out. These are all important learning points for both future researchers and device manufacturers. Progressive training on the surgical procedure and manufacture of more robust devices will probably mitigate some of these AEs in the future.

Our recordings had overall high adherence, higher than reported in a previous 3‐month trial.^28^ At the group level, we did not see a significant attrition rate in recording adherence, contrary to what is often reported in device studies.^38^ Half of our cohort had excellent adherence (>20 h/day). This shows that at least a proportion of patients are highly motivated to use these devices during their daily lives. Nevertheless, there were patients with poor adherence to the device. This might reflect periods around device deficiency, some AEs, the inconvenience and possible stigma of wearing a data logger, or the lack of perceived direct clinical benefit during this observational study. These points highlight the need to develop and improve devices that are less obtrusive, less stigmatizing, and above all that translate to a direct clinical benefit to the patient.^14^ Participants also showed individual patterns of adherence, at both circadian and weekly levels. This unbalanced adherence is important to consider, particularly when interpreting temporal trends of both seizures and interictal activity.

When comparing sqEEG seizures with diary events, expected discrepancies were encountered. Patients failed to report more than half of recorded seizures. One third of tonic–clonic sqEEG seizures were not associated with a diary report. This level of underreporting is consistent with previous studies comparing seizure diaries against video‐EEG^1^ and ambulatory EEG.^19^, ^39^ Reasons for underreporting have not been thoroughly investigated. Patients may lack the perception to report a seizure (e.g., seizure with impaired awareness and no warning or subjective symptoms), may forget a seizure occurred (e.g., seizure with retrograde amnesia), or may be unable to document it (e.g., due to temporary cognitive or motor impairment). Patients with particularly high seizure frequency may lose interest and motivation to track all seizures.

Conversely, 28% of diary reports were not associated with a recorded sqEEG seizure. The two‐channel unilateral device may have limited spatial sampling to detect some distant and/or contralateral seizures. Most nonrecorded diary events were reported as aware seizures, for which even full‐scalp EEG has limited sensitivity.^40^ Some seizures recorded in the real‐world may be obscured by activities of daily living and associated movement/muscle artifacts (examples in Viana et al.^27^). Importantly, overreporting is an often overlooked but clinically relevant aspect in diary seizure misdocumentation. Reasons may include the reporting of psychogenic nonepileptic seizures or of other paroxysmal events unrelated to seizures (e.g., cardiogenic syncope, panic attacks).

In this study, an automated seizure detection algorithm was used to reduce the amount of data to be reviewed to facilitate and accelerate the seizure annotation process. Across participants, the algorithm's sensitivity was well within the reported range for most scalp EEG‐based seizure detection algorithms (median of 88.8% against a reported range of 75%–90%),^41^ with also an acceptable prereview false detection rate (median of 3.4/day against a reported range of 2.4–120/day).^41^ The algorithm had excellent sensitivity in a proportion of participants, enabling significant reduction of the amount of data reviewed manually. However, sensitivity was low in a few patients, for whom comprehensive manual review was eventually needed (Supporting Information S17). These examples highlight a clear need for the improvement of automated seizure detection methods, particularly applied to limited channel real‐world EEG data. Future directions may include the incorporation of patient‐specific/personalized seizure patterns, well described in scalp^42^ and subcutaneous EEG data.^27^, ^43^ Initial triaging by technical staff is a potential approach that warrants further investigation.

One advantage of using sqEEG for seizure counting is to characterize different seizure types within the same patient, allowing phenotyping and risk stratification. Seizures with tonic–clonic artifacts were easily distinguishable from nonconvulsive seizures (Supporting Information S8), and good discrimination based on single‐channel surface electromyographic electrodes has been previously described.^44^ Further more refined characterization of seizure types, based on patient‐specific artifact patterns^27^ or seizure duration,^45^ may be possible with two‐channel data and should be the subject of future work.

Chronic EEG recordings have enabled the characterization of temporal dynamics of seizure occurrence at the individual level.^33^, ^34^, ^46^ Similarly to previous reports, we have observed individualized patterns of both seizure clustering and circadian periodicity. The proportion of clustered seizures compared to lead seizures varied widely between patients, from 2% to 87%, and the degree of clustering at different timescales showed high interindividual variability, but apparently high intraindividual stability (Figure 3). In addition, many seizure clusters were missed by seizure diaries, where often only one seizure was reported (examples in Figure 2). Identifying seizure clusters may have additional utility to prompt administration of short‐acting medication, particularly if the detection is timely.^36^

We have shown that it is also possible to determine individual circadian seizure periodicities using sqEEG, similar to other work with subscalp EEG.^47^ Circadian periodicity was very common in our cohort. It is interesting to note that, even though more than half of sqEEG seizures were not reported in the diary, in many patients the shape of the distribution of seizure periodicities between both modalities was similar. Nevertheless, several periodicities were only significant on sqEEG, whereas one was found only in diary data. These discrepancies may be due to biased misreporting in the diary (examples shown in Figure 2). Alternatively, they may arise from discontinuities in the recordings, which could also be biased for certain times of day. Forecasting performance has been shown to improve when circadian periodicity is included in prediction models.^48^ Future studies should further explore the feasibility and clinical utility of including sqEEG seizure circadian periodicity information in clinical practice. One option would include displaying polar plots in sqEEG reports (examples in Figure 3) for patients to be aware and for clinicians to consider adjusting medication timing.

This study has other limitations in addition to those reported above. The sample size is small and is not necessarily representative of the population of people with treatment‐resistant epilepsy. The exclusion criteria were wide in this study, which may have affected a more widespread recruitment. The placement of the subcutaneous EEG implant was determined after examining the patients' previous video‐EEG investigations, but no period of simultaneous video‐EEG and sqEEG was performed. We found it unfeasible to perform this in our study due to clinical demand for prolonged video‐EEG at our center and the likely need for medication reduction in some patients to capture seizures within a conventional video‐EEG study timeframe (e.g., the mean seizure frequency in SH was 2/month). Reducing antiseizure medication purely for research reasons would be ethically challenging given the associated risks. Furthermore, given the substantial seizure annotation task in this study, we did not assess agreement among different raters to identify sqEEG seizures.

A significant challenge will be to determine how to both implement and deliver this service within a health care system. Review of event detections is time consuming and requires specific expertise. After review, there will need to be a feasible way to relay the information back to both the patient and the clinician. This could take the form of a periodic report, for example, comparing a recent period with a reference. How frequently the information should be updated and shared remains to be determined.

The question arises as to which patients may benefit most from sqEEG as it is currently performed. Based on our findings and previous literature,^50^ sqEEG may be particularly valuable for patients with drug‐resistant focal epilepsy, detectable electrographic seizures, and suspected diary inaccuracy—such as those with significant postictal amnesia, nocturnal seizures, absence of witness reports, or those living alone. It may also be considered in patients where personalized treatment adjustments or chronotherapy‐based interventions could be beneficial.

In conclusion, we demonstrated that ultra‐long‐term subcutaneous EEG recordings are feasible and may provide a range of clinically useful information to patients with drug‐resistant epilepsy and their clinicians. It is possible to detect seizures more objectively, during routine daily life out of hospital. sqEEG can also help validate or contribute to the differential diagnosis of patients' symptoms. Recorded seizures can be categorized into different subtypes that, together with other EEG biomarkers, could allow more objective and timely disease severity stratification. It is possible to monitor individual temporal fluctuations of seizure occurrence, including circadian seizure periodicity, which could be highly valuable for patients to improve uncertainty. In addition, pending further validation, sqEEG may assist in treatment adjustment over time, helping to tailor the most effective medication load. Overall, these findings, conducted in a small group of patients but over a long time period, show the potential of sqEEG for a diverse range of clinical applications. This work calls for future, larger scale, prospective trials to further validate this technology.

## AUTHOR CONTRIBUTIONS

*Conception and design of the study:* Pedro F. Viana, Jonas Duun‐Henriksen, Dean R. Freestone, Andreas Schulze‐Bonhage, Benjamin H. Brinkmann, and Mark P. Richardson. *Acquisition and analysis of data:* Pedro F. Viana, Jonas Duun‐Henriksen, Andrea Biondi, and Joel S. Winston. *Manuscript draft:* Pedro F. Viana.

## CONFLICT OF INTEREST STATEMENT

J.D.‐H. is an employee of UNEEG Medical. DF is a shareholder of Seer Medical. B.H.B. has equity in Cadence Neurosciences, has received research devices from Medtronic at no cost, and has received research support from UNEEG and Neurelis. M.P.R. has been a member of ad hoc advisory boards for UNEEG Medical. P.F.V. has received travel and consultancy fees from UNEEG Medical. None of the other authors has any conflict of interest to disclose. We confirm that we have read the Journal's position on issues involved in ethical publication and affirm that this report is consistent with those guidelines.

## TRIAL REGISTRATION

NCT04061707.

## Supporting information


Data S1.

## Data Availability

Data from selected subcutaneous EEG recordings that support these findings will be made available in a future open‐source seizure prediction challenge.
